# Transcatheter closure of a posterior ascending aortic pseudoaneurysm after aortic dissection repair: a case report

**DOI:** 10.3389/fcvm.2025.1682988

**Published:** 2026-01-12

**Authors:** Phuc Nang Vu, Thuy Thuc Minh Pham, Thong Minh Luong, Binh Thanh Huynh, Vinh Nguyen Pham, Hieu Lan Nguyen

**Affiliations:** 1 Tam Anh General Hospital, Ho Chi Minh City, Vietnam; 2 School of Medicine, University of Medicine and Pharmacy at Ho Chi Minh City, Ho Chi Minh City, Vietnam; 3 Trung Vuong Hospital, Ho Chi Minh City, Vietnam; 4 Hanoi Medical University Hospital, Ha Noi, Vietnam

**Keywords:** aortic dissection repair, ascending aortic pseudoaneurysm, multimodality imaging, posterior pseudoaneurysm, septal occluder device, transcatheter closure

## Abstract

**Background:**

Ascending aortic pseudoaneurysm is a rare but potentially fatal complication after aortic surgery, particularly challenging in elderly high-risk patients.

**Case presentation:**

We report an 80-year-old man with a history of surgical repair for acute type A aortic dissection who presented with a two-month history of chest pain. Transthoracic echocardiography and computed tomography angiography revealed a large posterior ascending aortic pseudoaneurysm measuring 6.5 × 5.4 cm with a narrow neck. Because of his advanced age, previous sternotomy, and overall frailty, the patient was considered unsuitable for redo surgery after heart team discussion.

**Management and outcome:**

After obtaining written informed consent, the patient underwent successful percutaneous closure using a 10-mm Cocoon Septal Occluder device via transfemoral access. The procedure was uncomplicated, and immediate angiography confirmed minimal residual flow. Six-month follow-up computed tomography angiography demonstrated complete thrombosis of the aneurysm sac, and the patient remained asymptomatic with no residual shunt at two-year follow-up.

**Conclusion:**

This case illustrates that transcatheter closure of a posterior ascending aortic pseudoaneurysm using a septal occluder device can be a safe and durable alternative to high-risk reoperation, provided that careful pre-procedural imaging and catheter selection are performed.

## 1 Introduction

Aortic pseudoaneurysm (AAP) is a rare but potentially fatal complication after cardiovascular surgery, with an incidence of less than 0.5% among all cardiac operations (1). Posterior ascending aortic pseudoaneurysms are exceedingly rare and are particularly difficult to access due to their deep retrocardiac orientation and unfavorable trajectory from femoral access. Reoperation remains the standard of care but carries substantial morbidity and mortality, particularly in elderly patients or those with prior sternotomy. However, our successful management of an 80-year-old man with a posterior ascending aortic pseudoaneurysm using an atrial septal occluder device demonstrates the potential of transcatheter intervention as a less invasive alternative.

Posterior AAPs are exceptionally uncommon and technically challenging due to their deep retrocardiac location and the complex curvature introduced by femoral access. Only isolated reports have described transcatheter closure in such cases (2, 3). This case highlights an unusual anatomic presentation and a tailored interventional strategy, underscoring the importance of multimodality imaging, wire handling, and device selection.

## 2 Case report

The patient was an 80-year-old man admitted with a two-month history of substernal chest pain. Seven years earlier, he had undergone surgical repair of an acute type A aortic dissection using a 26-mm Gore-Tex graft. He also had a history of hypertension and dyslipidemia. On physical examination, findings were unremarkable. However, considering his advanced age and previous major cardiac surgery, his general condition was considered frail.

**Figure 1:**
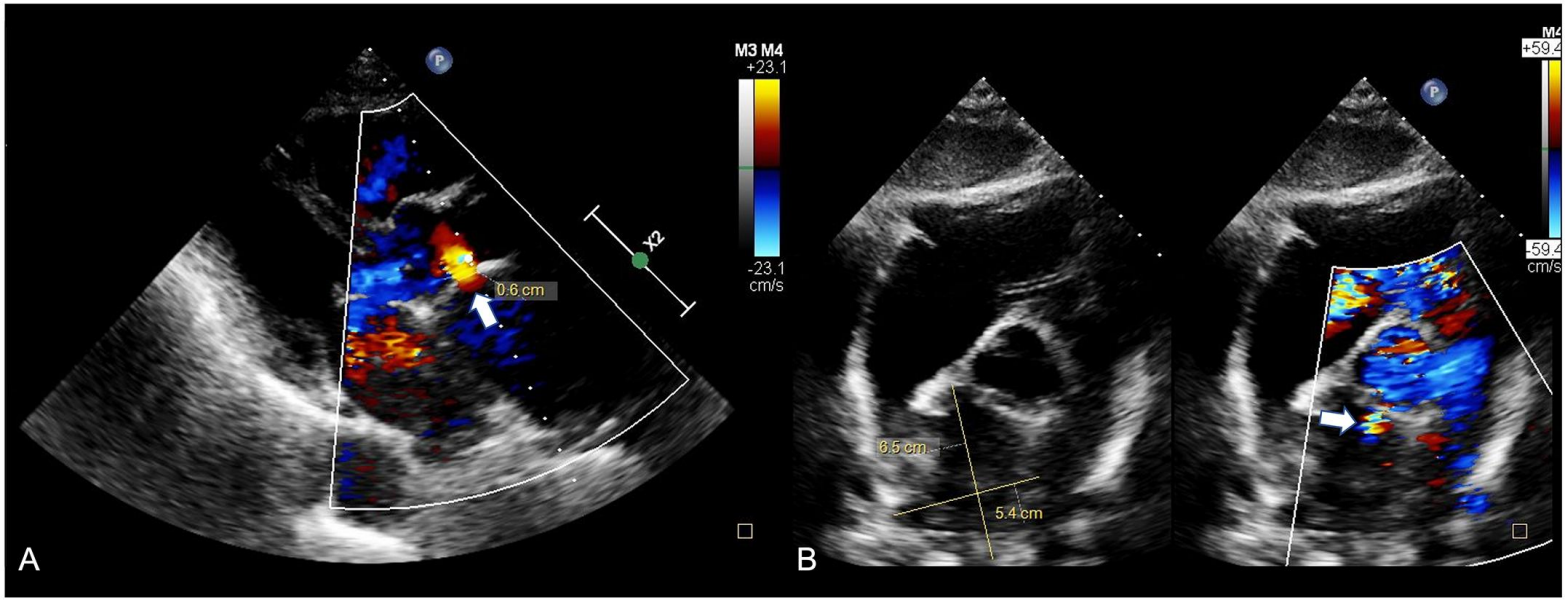
Modified subcostal short-axis view **(A)** and PLAX view **(B)** with a 6.5 × 5.4 cm AAP with the neck just above the non-coronary cusp of the aortic valve.

**Figure 2:**
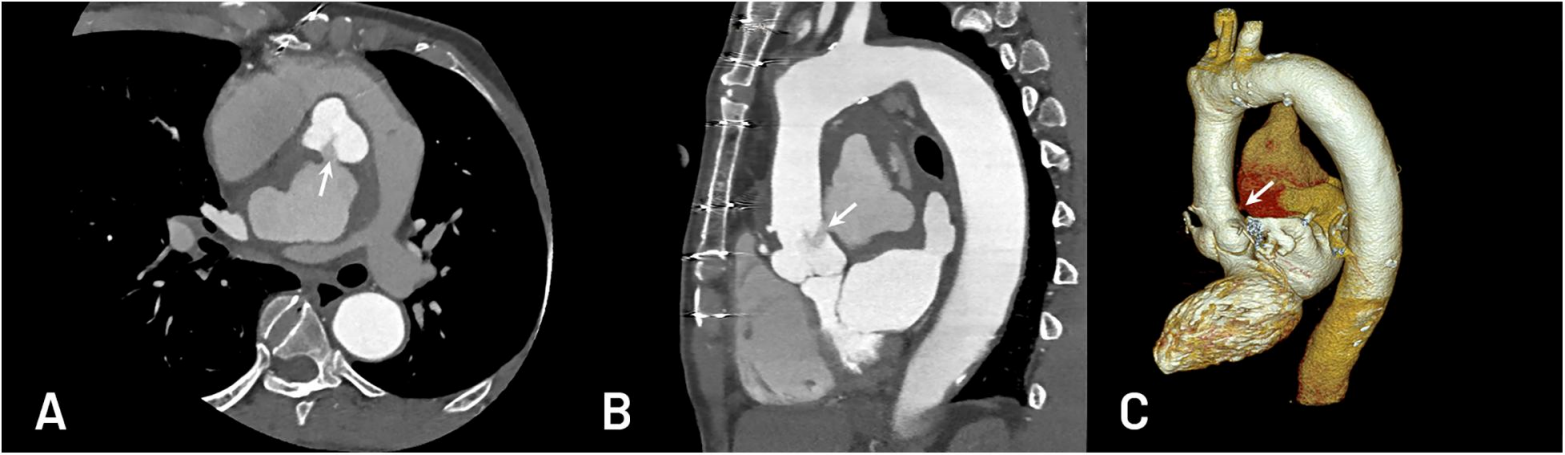
CTA showed a giant posterior ascending aortic pseudoaneurysm with a 7 mm neck **(A)** above the non-coronary cusp and partially compressed the left atrium and right pulmonary artery **(A,B)**. 3D reconstruction of pseudoaneurysm **(C)**.

Transthoracic echocardiography (TTE) revealed a 6.5 × 5.4 cm AAP located posterior to the sinotubular junction of the ascending aorta. The pseudoaneurysm neck, measuring 6 mm, originated from the proximal anastomosis between the native aorta and the graft, with bidirectional flow between the aorta and the pseudoaneurysm cavity (Figures 1A,B). Computed tomography angiography (CTA) confirmed a giant posterior AAP approximately 6.0 × 6.0 cm in size, with a 7 mm neck that partially compressed the left atrium and right pulmonary artery (Figures 2A,B). Three-dimensional reconstruction demonstrated that the defect was above the non-coronary cusp and distant from the coronary orifices (Figure 2C). No evidence of infection or endocarditis was found. Because of his frailty, previous sternotomy, and high operative risk, the heart team decided on a percutaneous approach after obtaining written informed consent.

Under local anesthesia, bilateral femoral access was obtained using 8 Fr and 6 Fr short sheaths to allow simultaneous angiography and device delivery. The patient was heparinized with 5,000 units of unfractionated heparin to maintain an activated clotting time above 200 s. Ascending aortography confirmed the diagnosis and defined the optimal working projection (left anterior oblique 60 °) for visualization of the pseudoaneurysm neck.

**Figure 3:**
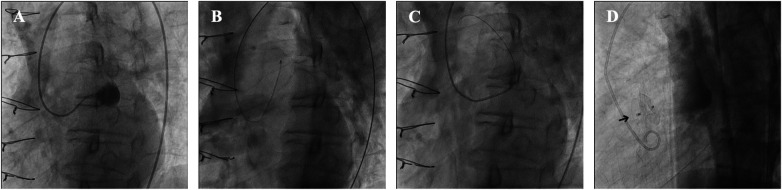
Transcatheter closure of ascending aortic pseudoaneurysm (AAP) after cardiac surgery. **(A)** Selective engagement of the AAP with a 5F Amplatz Left-1 diagnostic catheter; contrast injection confirmed no coronary involvement. **(B)** Amplatz Super Stiff™ wire was inserted into the pseudoaneurysm, and the patient had chest pain and hypotension. **(C)** The AAP was engaged with a 5F Judkins Right 4.0 catheter, followed by an exchange for a 0.035” Radifocus Guide Wire M Standard Type. **(D)** A 10-mm Cocoon Septal Occluder (arrowhead) was successfully deployed across the defect.

Under fluoroscopic guidance, the AAP was selectively engaged with a 5F Optitorque® Amplatz Left-1 catheter (Terumo, Tokyo, Japan). A small contrast injection confirmed that the coronary ostia were not involved (Figure 3A). A 0.035-inch hydrophilic Radifocus® guidewire (Terumo, Tokyo, Japan) was advanced into the pseudoaneurysm cavity, followed by cautious exchange for a 260-cm Amplatz Super Stiff™ wire (Boston Scientific, Natick, MA, USA) for better support. During manipulation, transient chest pain and hypotension (systolic blood pressure 85 mmHg) occurred, likely due to tension at the pseudoaneurysm neck; symptoms resolved promptly after the stiff guidewire was withdrawn (Figure 3B).

To minimize the risk of rupture, the team switched to a softer 0.035-inch Fixed Core J-tip guidewire (Argon, Frisco, TX, USA) supported by a Judkins Left 4.0 catheter. An 8 Fr Launcher™ right coronary curve guiding catheter (Medtronic, Minneapolis, MN, USA) was then advanced into the pseudoaneurysm (Figure 3C). A 10-mm Cocoon Septal Occluder (Vascular Innovations, Nonthaburi, Thailand) was successfully deployed across the 7-mm neck (Figure 3D), with careful attention to avoid deep advancement or coronary compression.

Final aortography confirmed correct device position and nearly complete cessation of flow into the pseudoaneurysm. The procedure was uneventful, and the patient recovered hemodynamically stable. He was discharged after three days with complete symptom resolution.

Follow-up imaging demonstrated excellent procedural results. Six months after the intervention, CTA showed complete thrombosis of the pseudoaneurysm cavity without contrast enhancement or increase in sac size, confirming successful exclusion of the lesion. The patient remained asymptomatic, with stable echocardiographic findings and no residual flow or shunt.

During two years of clinical follow-up, he remained well without recurrence, device migration, or new aneurysm formation.

## 3 Discussion

AAP is an uncommon but serious complication that may occur after cardiovascular or mediastinal surgery, with an incidence of approximately 0.5% among all cardiac operations (1). It can appear soon after surgery or many years later, even decades after the first operation (4). Early morbidity and mortality have been reported at around 20%, and the annual mortality may reach 40% if the lesion is left untreated (5). Because of its natural tendency to enlarge and rupture, surgical repair is usually required, although reoperation is technically demanding and carries substantial risk (6–8). Possible causes include intrinsic aortic wall weakness, suture dehiscence, or perioperative infection, which gradually weaken the anastomotic site and promote pseudoaneurysm formation. As the cavity enlarges, it can compress or erode surrounding structures, rupture, create fistulous connections, or lead to thrombosis and death.

The diagnosis of AAP is often delayed because symptoms are nonspecific or absent. Echocardiography remains a key tool for detection, especially when sequential studies show new abnormal flow or cavity formation. In our patient, TTE revealed a large posterior pseudoaneurysm, a location that can be easily missed due to acoustic shadowing and surgical graft artifacts. Lowering the color Doppler velocity scale helped identify the bidirectional flow between the ascending aorta and the pseudoaneurysm, particularly the diastolic flow returning from the sac into the aorta (Figure 1). A modified subcostal short-axis view also confirmed the neck and its relation to the graft anastomosis.

CTA was essential for confirming the diagnosis and planning the procedure. It clearly demonstrated the neck size, spatial relationship with the coronary arteries, and the degree of compression on adjacent structures (Figure 2). Combining echocardiography and CTA enabled an accurate assessment of both hemodynamics and anatomy, providing complementary information for intervention planning.

Surgical reintervention remains the standard treatment for ascending aortic pseudoaneurysm, yet it is technically complex and associated with significant perioperative mortality (6–8). For patients who are deemed inoperable or at prohibitive surgical risk, transcatheter closure offers a feasible alternative (2). Over the past two decades, several endovascular methods have been reported, including direct thrombin injection, coil embolization, covered stent grafting, and occlusion with septal or vascular plug devices (2, 8). The first successful case using an Amplatzer septal occluder for ascending aortic pseudoaneurysm was published in 2005 (3), followed by multiple reports confirming its safety and long-term efficacy (8, 9).

Most previously reported cases involved anterior or lateral pseudoaneurysms, which are easier to engage from the femoral approach. In contrast, our case involved a posterior lesion requiring navigation through two sharp angulations, which made catheter positioning and device delivery more challenging. A Judkins Left or Amplatz Left catheter can provide adequate support to reach the neck, but the use of a stiff guidewire may distort the aortic root or precipitate rupture. In our patient, transient chest pain and hypotension occurred during stiff-wire manipulation, which resolved after the wire was withdrawn. This experience emphasizes that gentle wire handling and timely conversion to a soft guidewire are critical to avoid procedural complications. To our knowledge, a posterior AAP has not been previously reported. This posterior location required a tailored interventional strategy under multimodality imaging guidance.

A guiding catheter with appropriate curvature and flexibility allows safe advancement of the occluder device without excessive force. Careful positioning and gradual deployment under fluoroscopic and angiographic control are essential to prevent migration or compression of nearby structures. The successful outcome in this case supports the role of individualized catheter selection, multimodality imaging guidance, and cautious technique in achieving a durable result.

Device selection and procedural considerations are essential when planning transcatheter repair of an ascending aortic pseudoaneurysm. The choice of occlusion device depends largely on the neck morphology, surrounding tissue stability, and the relationship to adjacent structures. Although a septal occluder was appropriate in our patient because of the relatively narrow and well-defined neck, other devices—such as duct occluders or vascular plugs—may be more suitable when the neck is tubular, wider, or less rigid. Conversely, anatomy with a huge neck, extremely thin or friable tissue, or suspicion of infection is generally unsuitable for percutaneous closure, and surgical repair remains the preferred management strategy in such scenarios.

Access selection also requires careful consideration, especially for posterior pseudoaneurysms. Alternative approaches, such as the right brachial or even the carotid artery, can theoretically provide a more direct and favorable trajectory to the ascending aorta, thereby reducing catheter angulation and improving coaxial alignment with the pseudoaneurysm neck. However, these routes are substantially limited by their ability to accommodate large delivery sheaths, which are often required for septal occluder or vascular plug systems. Moreover, upper-extremity or carotid access carries higher risks of vascular complications, including arterial injury, hematoma, and neurologic events. It is less commonly used in routine structural interventions, resulting in reduced operator familiarity compared with the transfemoral route.

In our case, transfemoral access remained the most practical and safest option for an 8 Fr delivery system, despite the challenging posterior orientation. With meticulous wire manipulation and catheter support, adequate stability was achieved without excessive tension at the pseudoaneurysm neck.

This case highlights several important lessons for clinical practice. A posterior ascending aortic pseudoaneurysm is technically demanding both to diagnose and to treat. Transthoracic echocardiography can miss such lesions unless the operator intentionally lowers the color Doppler scale and inspects the anastomotic region in multiple planes. Computed tomography complements echocardiography by defining the exact anatomy, neck orientation, and relationship to surrounding structures, all of which are critical for planning a safe percutaneous approach.

From a procedural standpoint, this case demonstrates that avoiding excessive stiffness in the guidewire and selecting a catheter with appropriate curvature can minimize the risk of rupture. The successful exclusion of the pseudoaneurysm and sustained clinical stability during long-term follow-up suggest that, in carefully selected high-risk patients, transcatheter closure with a septal occluder may serve as a durable, less invasive alternative to reoperation (2, 3, 8, 9).

Ultimately, this experience underscores the value of multimodality imaging, heart-team discussion, and tailored interventional strategy in managing complex post-surgical aortic complications.

## 4 Conclusion

Posterior ascending aortic pseudoaneurysm is an uncommon but critical complication that requires a high index of suspicion and careful imaging assessment. When surgery carries excessive risk, transcatheter closure using a septal occluder device can provide a safe and effective alternative, provided that the anatomy is well defined and the procedure is performed under experienced guidance. Meticulous wire and catheter manipulation are essential to avoid rupture or device instability. This case illustrates that a patient-specific, imaging-guided strategy can achieve durable results in situations where conventional surgery is not feasible.

## Data Availability

The original contributions presented in the study are included in the article/Supplementary Material, further inquiries can be directed to the corresponding author.

## References

[1] Mesana TG Caus T Gaubert J Collart F Ayari R Bartoli J Late complications after prosthetic replacement of the ascending aorta: what did we learn from routine magnetic resonance imaging follow-up? Eur J Cardiothorac Surg. (2000) 18(3):313–20. 10.1016/S1010-7940(00)00512-1 10973541

[2] Quevedo HC Santiago-Trinidad R Castellanos J Atianzar K Anwar A Abi Rafeh N. Systematic review of interventions to repair ascending aortic pseudoaneurysms. Ochsner J. (2014) 14(4):576–85. PMCID: PMC4295735 PMID: 25598723. 25598723 PMC4295735

[3] Kanani RS Neilan TG Palacios IF Garasic JM. Novel use of the amplatzer septal occluder device in the percutaneous closure of ascending aortic pseudoaneurysms: a case series. Catheter Cardiovasc Interv. (2007) 69(1):146–53. 10.1002/ccd.20794 17139656

[4] Konia M Uppington J Moore P Liu H. Ascending aortic pseudoaneurysm: a late complication of coronary artery bypass. Anesth Analg. (2008) 106(3):767–8. 10.1213/ane.0b013e3181608bbd 18292416

[5] Carrel T Pasic M Jenni R Tkebuchava T Turina MI. Reoperations after operation on the thoracic aorta: etiology, surgical techniques, and prevention. Ann Thorac Surg. (1993) 56(2):259–68. 10.1016/0003-4975(93)91157-I 8347007

[6] Sullivan KL Steiner RM Smullens SN Griska L Meister SG. Pseudoaneurysm of the ascending aorta following cardiac surgery. Chest. (1988) 93(1):138–43. 10.1378/chest.93.1.138 3257182

[7] Mulder EJ van Bockel JH Maas J van den Akker PJ Hermans J. Morbidity and mortality of reconstructive surgery of noninfected false aneurysms detected long after aortic prosthetic reconstruction. Arch Surg. (1998) 133(1):45–9. 10.1001/archsurg.133.1.45 9438758

[8] Lyen SM Rodrigues JC Manghat NE Hamilton MC Turner M. Endovascular closure of thoracic aortic pseudoaneurysms: a combined device occlusion and coil embolization technique in patients unsuitable for surgery or stenting. Catheter Cardiovasc Interv. (2016) 88(7):1155–69. 10.1002/ccd.26558 27141915

[9] Mussa FF LeMaire SA Bozinovski J Coselli JS. An entirely endovascular approach to the repair of an ascending aortic pseudoaneurysm. J Thorac Cardiovasc Surg. (2007) 133(2):562–3. 10.1016/j.jtcvs.2006.10.010 17258601

